# Assessment at Antiretroviral Clinics during TB Treatment Reduces Loss to Follow-Up among HIV-Infected Patients

**DOI:** 10.1371/journal.pone.0037634

**Published:** 2012-06-18

**Authors:** Dominique J. Pepper, Suzaan Marais, Feriyl Bhaijee, Robert J. Wilkinson, Virginia De Azevedo, Graeme Meintjes

**Affiliations:** 1 Department of Medicine, Clinical Infectious Diseases Research Initiative, Institute of Infectious Diseases and Molecular Medicine, University of Cape Town, Western Province, South Africa; 2 Department of Medicine, University of Mississippi Medical Center, Jackson, Mississippi, United States of America; 3 Department of Medicine, Infectious Diseases Unit, GF Jooste Hospital, Cape Town, South Africa; 4 Division of Medicine, Imperial College London, London, United Kingdom; 5 MRC National Institute for Medical Research, London, United Kingdom; 6 City Health, Cape Town, South Africa; Hannover Medical School, Germany

## Abstract

**Setting:**

A South African township clinic where loss to follow-up during TB treatment may prevent HIV-infected TB patients from receiving life-saving ART.

**Objective:**

To determine factors associated with loss to follow-up during TB treatment.

**Design:**

Regression analyses of a cohort of ART-eligible TB patients who commenced TB treatment and were followed for 24 weeks.

**Results:**

Of 111 ART-eligible TB patients, 15 (14%) died in the ensuing 24 weeks. Of the remaining 96 TB patients, 11 (11%) were lost to follow-up. All TB patients lost to follow-up did not initiate ART. Of 85 TB patients in follow-up, 62 (73%) initiated ART 56 days after TB diagnosis (median, IQR 33–77 days) and 31 days after initial assessment at an ART clinic (median, IQR: 18–55 days). The median duration from TB diagnosis to initial assessment at an ART clinic was 19 days (IQR: 7–48 days). At 24 weeks, 6 of 85 (7%) TB patients who presented to an ART clinic for assessment were lost to follow-up, compared to 5 of 11 (45%) TB patients who did not present to an ART clinic for assessment. Logistic regression analysis (adjusted odds ratio  = 0.1, 95% confidence interval [95% CI]: 0.03–0.66) and our Cox proportional hazards model (hazard ratio  = 0.2, 95% CI: 0.04–0.68) confirmed that assessment at an ART clinic during TB treatment reduced loss to follow-up.

**Conclusion:**

Assessment at antiretroviral clinics for HIV care by trained health-care providers reduces loss to follow-up among HIV-infected patients with TB.

## Introduction

In sub-Saharan Africa, tuberculosis (TB) is the most frequent cause of death in HIV-1 infected adults [1]: between 8% and 23% of HIV-infected TB patients die during TB treatment. [2], [3] The initiation of antiretroviral treatment (ART) during TB treatment among those with severe immune-suppression improves survival. [4] In HIV-infected TB patients with CD4 counts less than 50 cells/microL, initiation of ART 1–3 weeks after commencing TB treatment reduces mortality and/or the development of AIDS by 34–68%, compared to initiating ART later during TB treatment. [5]–[7].

Thus, timely initiation of ART during TB treatment is a priority. However, TB patients co-infected with HIV typically attend completely separate HIV and TB clinics in different localities. [8] Up to 9% of HIV-infected TB patients are lost to follow-up during TB treatment, [9], [10] precluding initiation of ART. Identifying those at risk of loss to follow-up is essential. In this study, we performed a secondary analysis of a recently described prospective cohort of HIV-infected TB patients [11], [12] in order to determine factors associated with loss to follow-up during TB treatment.

## Methods

### Study Population

We conducted our study in a high density (>7500 inhabitants/km^2^), predominantly black African township in South Africa, [13] where annual TB case notification rates approach 1,600/100,000 people of the general population. TB patients in this township are treated in TB clinics administered by Cape Town’s Health Department. According to national protocol, TB patients receive standardized TB treatment regimens using Directly Observed Therapy Short-course (DOTS). [14] National guidelines at the time of our study recommended ART for all TB patients with a CD4+ cell count less than 200 cells/µL or a history of a WHO stage 4 illness. [14] Extra-pulmonary TB – although a World Health Organisation (WHO) stage 4 illness – was not an indication for ART unless the patient’s CD4+ count was less than 200 cells/µL. First-line ART during our study was stavudine, lamivudine, and either nevirapine or efavirenz. Efavirenz was preferred for adults receiving rifampin-based TB treatment. National guidelines also recommended daily trimethoprim-sulfamethoxazole (160/800mg) chemoprophylaxis. [14].

Our study center is one of the first in South Africa to successfully integrate HIV and TB healthcare services. As a result, our TB cohort is characterised by high rates of i) voluntary counselling and testing of HIV status (>95%), ii) rigorous testing of CD4+ counts if HIV-infected (>99%), and iii) provision of trimethoprim-sulfamethoxazole chemoprophylaxis (>95%). [11] Moreover, DOTS coverage is >80% at this center (personal communication – Judy Caldwell, Cape Town Health Department).

We have previously described our prospective cohort of 209 HIV-infected TB patients (≥18 years of age), which we recruited at our study center. Data obtained from this cohort was used to determine the incidence, risk factors, and causes of clinical deterioration during 6 months of TB therapy, [10] as well as identify barriers to initiation of ART during TB treatment. [11] All adults in our cohort were recruited at initiation of TB therapy – regardless of HIV status – and followed for 6 months. Written informed consent was obtained from enrolled adults and the Research Ethics Committee of the University of Cape Town approved this study (REC 178/2008).

**Figure 1:**
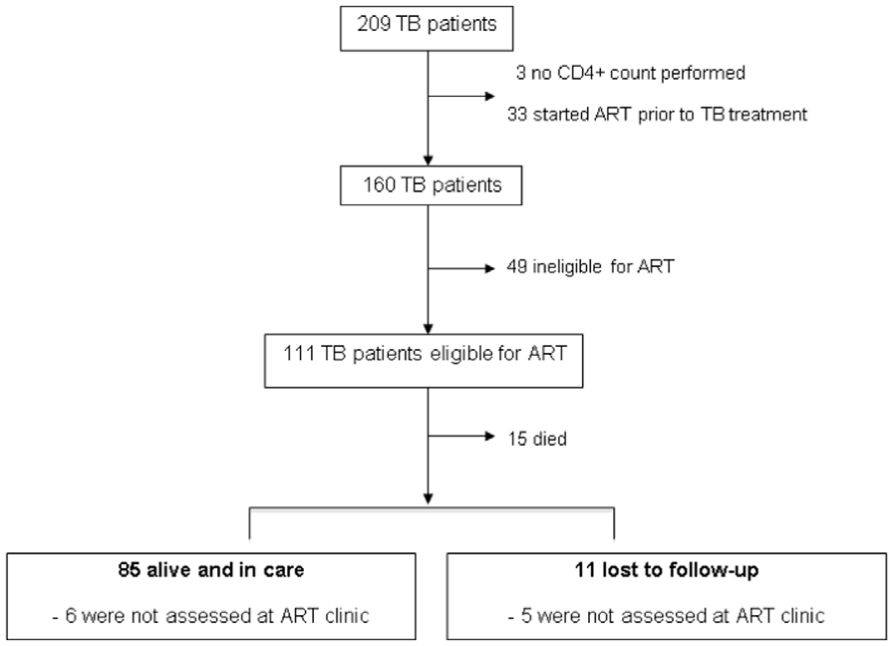
Flow-diagram showing inclusion criteria and outcomes of 111 patients eligible for antiretroviral treatment at TB diagnosis. ART: antiretroviral treatment, TB: tuberculosis.

The following is a secondary analysis of this cohort: among those eligible to receive ART, we determined factors associated with loss to follow-up. Of 209 enrolled HIV-infected TB patients (figure 1), 111 comprised our study population as they were eligible to initiate ART at TB diagnosis according to national guidelines. Reasons for excluding the remaining 98 TB patients are shown in figure 1: CD4+ count not performed (n = 3), ART started prior to TB treatment (n = 33), transferred out (n = 13), and ineligible for ART as CD4+ count greater than 200 cells/µL (n = 49). Of the 111 eligible adults (figure 1), 15 (14%) died, 11 (10%) were lost to follow-up, and 85 (76%) were alive and under our care at 24 weeks of follow-up. We defined ‘transferred out’ as transfer of care to another tuberculosis clinic at a patient’s request. This transfer was facilitated by a written referral letter and resulted in exclusion from our study. We defined ‘lost to follow-up’ as being unable to trace a TB patient 24 weeks after commencing TB treatment. Patients not lost to follow-up were alive and under our care at 24 weeks of follow-up. We used clinic and hospital charts, as well as the Provincial Government of the Western Cape’s electronic tuberculosis register (ETR.net), [15] to trace TB patients and record clinical outcomes.

We determined the proportion of eligible TB patients lost to follow-up during TB treatment. In our setting, integration of TB and HIV services is the goal. As such, ART-eligible TB patients were referred with a written letter to their nearest ART clinic for assessment. TB patients who presented to the ART clinic for assessment received ART education from trained counsellors. After appropriate counselling and evaluation by either a nurse or doctor, TB patients initiated ART. In our study, a TB patient who attended one or more ART clinic appointment(s) was considered to have ‘presented to an ART clinic for assessment.’ We reviewed TB patients’ hospital and ART charts, the Western Cape’s electronic tuberculosis register (ETR.net) [15] and Cape Town’s electronic eKapa ART database to record those who presented to an ART clinic for assessment, as well as those who initiated ART.

We defined clinical deterioration as symptomatic worsening or failure to stabilise within 24 weeks following initiation of TB treatment. [11] Causes of clinical deterioration included AIDS-defining illnesses (according to WHO stage 4 criteria), non AIDS-defining HIV-related infections, TB-related illnesses, and illnesses unrelated to TB, i.e. co-morbid illnesses. These illnesses have been described in detail in a previous report. [11].

### Statistical Analysis

We performed statistical analysis using Stata 10.0 (Texas, USA). We found that age, weight at TB diagnosis, and duration from commencing TB treatment to ART initiation were right (non-normal) skewed but had normal distributions with logarithmic transformation (Shapiro-Wilk test); the mean age was used to dichotomise age categories (age <36 years vs age >36 years). Proportions were calculated for categorical variables and described using 95% confidence intervals (CI). We used Fisher’s exact test to determine which categorical variables were significantly associated with loss to follow-up. A p-value of less than 0.05 was considered significant.

Using logistic regression analysis, we explored relationships between significant variables and loss to follow-up. A backward stepwise logistic model was proposed to quantify these relationships, which were reported using odds ratios and 95% CI (table 1). We fitted the model using the likelihood ratio, which was logarithmically transformed to generate the chi-squared statistic. Those lost to follow-up had a shorter period of follow-up, so we created a Cox proportional hazards model to assess the independent effects of covariates. A backward stepwise model was proposed and variables were removed from the model to assess whether the effects remained (table 2). The assumptions of the Cox model were verified: censoring was non-informative and the tests for the proportional hazards assumption were not significant.

**Table 1:** Description of Tuberculosis Patients.

	n (%)	
Male gender	48	(50)
Age <36 years	56	(58)
CD4+ count <100 cells/µL	62	(65)
TMP-SMX chemoprophylaxis	91	(95)
Previous TB	25	(26)
Diagnosis of TB at hospital	46	(48)
Extra-pulmonary TB	41	(43)
Drug susceptibility test results known at TB diagnosis	26	(27)
Weight less than 50 kilograms	27	(28)
Assessed at ART clinic	85	(89)
ART initiated during TB treatment	62	(65)
Experienced clinical deterioration	55	(57)
Admission to hospital	39	(41)

LTF: loss to follow-up, ART: antiretroviral treatment, TB: tuberculosis, TMP-SMX chemoprophylaxis: daily trimethoprim sulfamethoxazole chemoprophylaxis 160/800mg.

**Table 2:** Univariate analyses and logistic regression model showing variables associated with loss to follow-up during TB treatment.

	OR	95% CI	aOR	95% CI
Age <36 years	1.23	(0.35–4.34)	1.75	(0.36–8.40)
TMP-SMX chemoprophylaxis	0.16	(0.02–1.12)	0.25	(0.02–2.80)
Extra-pulmonary TB	0.46	(0.12–1.87)	1.13	(0.20–6.48)
Drug susceptibility test results known at TB diagnosis	3.90	(1.08–14.2)	3.19	(0.63–16.2)
Assessed at ART clinic	0.09	(0.02–0.39)	0.14	(0.03–0.66)
ART initiated during TB treatment	0.01	(0.01–0.17)	–	–
Experienced clinical deterioration	0.38	(0.10–1.40)	0.61	(0.14–2.65)

ART: antiretroviral treatment, TB: tuberculosis, TMP-SMX chemoprophylaxis: daily trimethoprim sulfamethoxazole chemoprophylaxis 160/800mg.

For this logistic regression model: P = 0.023, R^2^ = 0.2147, ART initiation was a collinear variable so was omitted from analysis.

## Results

### Description of TB Patients

Among the 96 TB patients eligible for analysis, the mean age was 36 years (95% CI: 22–57 years) and 50% were male (Table 1). At TB diagnosis, 65% of TB patients had a CD4+ count <100 cells/µL, 26% had a previous history of TB, and 43% had extra-pulmonary TB (with or without co-existent pulmonary TB). In 27% of TB patients, results for TB drug susceptibility testing were known at TB diagnosis. During TB treatment, 95% of TB patients received trimethoprim-sulfamethoxazole chemoprophylaxis, 57% experienced clinical deterioration and 41% required hospital admission. 85 of 96 (89%) TB patients were alive and in care at completion of TB treatment, 79 of whom were assessed at an ART clinic within 19 days of TB diagnosis (median, interquartile range: 7–48 days). Eleven of 96 (11%) TB patients were lost to follow-up 68 days (median, interquartile range: 64–128 days) after commencing TB treatment; 5 of these patients were not assessed at an ART clinic and all did not initiate ART. Of the 85 TB patients in follow-up, 62 (73%) initiated ART 56 days after TB diagnosis (median, IQR: 33–77 days) and 31 days after initial assessment at an ART clinic (median, IQR:18–55 days). 23 of 85 (27%) TB patients in follow-up did not initiate ART. Overall, 34 of 96 (35%) eligible TB patients did not initiate ART during TB treatment.

### Risk Factors for Loss to Follow-up

**Figure 2:**
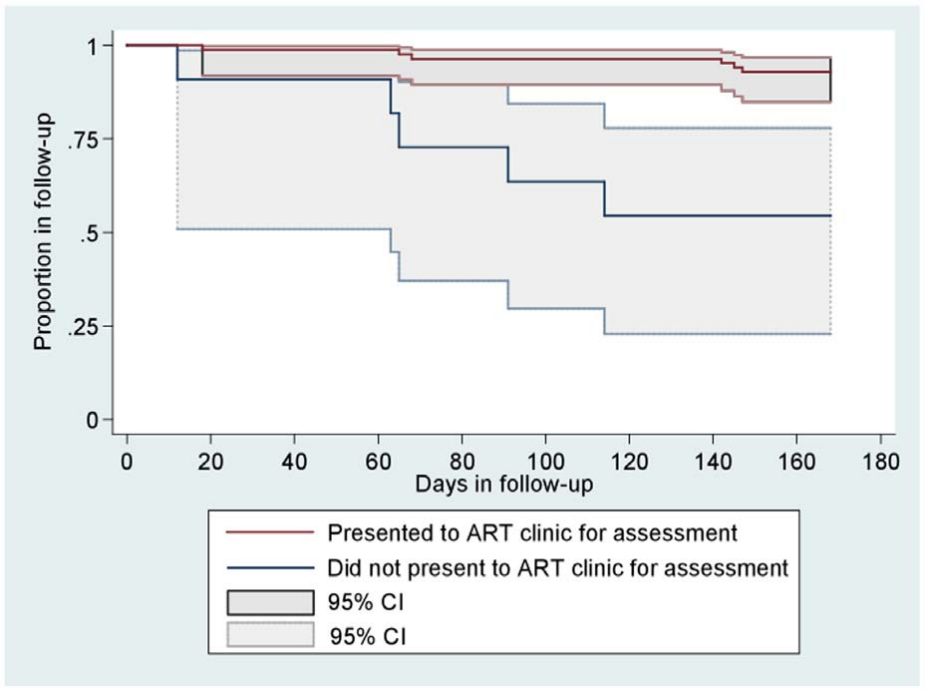
Kaplan-Meier estimates for remaining in follow-up during TB treatment, according to whether eligible adults presented to an antiretroviral clinic for assessment or not. ART: antiretroviral treatment.

At 24 weeks of follow-up, 7% (6/85) of adults who presented to an ART clinic for assessment were lost to follow-up, compared to 45% (5/11) of those who did not present to an ART clinic. In univariate analyses, the following were significantly associated with loss to follow-up: not receiving trimethoprim-sulfamethoxazole chemoprophylaxis, knowing the results for TB drug susceptibility testing at TB diagnosis, not presenting to an ART clinic for assessment, and not initiating ART. All TB patients who were lost to follow-up did not initiate ART, while 73% (62 of 85) of TB patients in follow-up initiated ART. Using our logistic regression model (P = 0.023, R^2^ = 0.2147), we found that presentation to an ART clinic for assessment was the only factor associated with decreased loss to follow-up (odds ratio  = 0.1, 95%CI: 0.03–0.66, table 2). Our Cox proportional hazards model (P = 0.025, Harrell’s C = 0.791, Somers’ D = 0.582) confirmed this significant association (hazard ratio  = 0.2, 95%CI: 0.04–0.68, table 3). Figure 2 is a Kaplan-Meier plot, which shows that from 12 weeks of follow-up onward, a significant and distinct trend emerged for loss to follow-up according to whether patients were assessed at an ART clinic or not.

**Table 3:** Cox Proportional Hazards Model for loss to follow-up during TB treatment.

	aHR	95% CI
Age <36 years	1.73	(0.44–6.74)
TMP-SMX chemoprophylaxis	0.33	(0.06–1.80)
Extra-pulmonary TB	0.97	(0.20–4.67)
Drug susceptibility test results known at TB diagnosis	2.17	(0.53–8.87)
Assessed at ART clinic	0.17	(0.04–0.68)
Experienced clinical deterioration	0.92	(0.22–3.84)

ART: antiretroviral treatment, TB: tuberculosis, TMP-SMX chemoprophylaxis: daily trimethoprim sulfamethoxazole chemoprophylaxis 160/800mg.

For this Cox proportional hazards model: P = 0.025, Harrell’s C = 0.791, Somers’ D = 0.582, ART initiation was a collinear variable so was omitted from analysis.

## Discussion

We conducted a retrospective analysis of a cohort of HIV-infected TB patients eligible for ART while receiving TB treatment. We found that loss to follow-up occurred in 11% of TB patients eligible for ART. We also found that assessment at an ART clinic during TB treatment reduced loss to follow-up. Our findings have important implications for TB and ART policy-makers in Africa: the trends we report suggest that interventions to reduce loss to follow-up should be implemented during TB treatment, preferably within the first three months. Our findings resonate with a recent South African study, which reported a trend in reduction of loss to follow-up among patients initiating ART at CD4 counts less than 200 cells/µL. [16].

Our findings are best interpreted in the following context: compared to other clinics in South Africa, the Khayelitsha Site B TB clinic benefits from strong partnerships that exist between the City of Cape Town, the Department of Health and the non-governmental organisation *Médecins Sans Frontieres*. The Site B TB clinic has received numerous accolades for pioneering the integration of HIV and TB services in South Africa. However, despite significant achievements, such as voluntary counselling and testing of HIV status in >95% of TB patients, drug susceptibility testing on almost all bacteriologic specimens during the study period, and trimethoprim-sulfamethoxazole chemoprophylaxis in >95% of HIV-1 infected patients, [11] loss to follow-up remains a significant challenge.

The substantial proportion of patients lost to follow-up (11%) is similar to reported rates in the international literature, in which up to 9% of HIV-infected patients are lost to follow-up during TB treatment. [9], [10] Among HIV-infected patients (with or without TB), loss to follow-up during ART is similar, varying between 2–13%. [17], [18] One of the strengths of our study is that we determined modifiable risk factors for loss to follow-up, which are typically difficult to ascertain. Using multivariate analysis, which incorporated a number of demographic, HIV, TB, and operational factors, we found that loss to follow-up was associated with i) not presenting to an ART clinic for assessment, and ii) not initiating ART (the latter in univariate analysis, which was subsequently excluded from our multivariate model due to collinearity). It is not known whether loss to follow-up resulted in patients not receiving ART, or whether the failure to initiate ART in these patients resulted in loss to follow-up. It is certainly plausible that ART initiation improves follow-up: in adults who suffer life-threatening illnesses due to profound immune-suppression, the initiation of ART not only improves immune function, survival, and well-being, but also promotes regular ART clinic attendance. Moreover, ART clinics offer an additional safety net of counselling and medical support. Other studies have demonstrated the benefit of co-treatment: ART with TB treatment increases retention during follow-up, compared to ART alone. [19] Likewise, in our study, we found that attending an ART clinic during TB treatment was independently associated with improved follow-up, compared to TB treatment alone.

We acknowledge that while we were able to determine risk factors for loss to follow up, we were not able to elucidate the underlying reasons for loss to follow up. In our setting, the following obstacles impair this investigation: i) most patients reside in informal housing (‘shacks’), the addresses of which are vulnerable to change with inclement weather and renovation; ii) cellular/mobile telephones are the preferred method of contacting adults in informal housing, but these telephones are subject to frequent exchange, theft and loss; and iii) the dynamic flux of people between Cape Town and the Eastern Cape Province (1000km eastward of Cape Town) hampers data collection, as patients may travel to the Eastern Cape Province and receive health care or die there. Future studies are needed to determine reasons for loss to follow-up. As described previously, twenty-four weeks of follow-up is a short period of observation. [11] It is possible that after 24 weeks, other factors may be associated with loss to follow-up. Of concern, a substantial proportion of TB patients (17/85, 20%) who presented to an ART clinic and remained in care did not initiate ART during TB treatment. Reasons for these delays also require further evaluation.

Furthermore, based on our findings, we have expanded our TB-ART care so that each HIV-infected patient with tuberculosis has one folder and is assessed at each appointment by a doctor or nurse who is skilled in the care of both diseases. This method has ensured that appropriate HIV care and assessment for ART is provided at the initial tuberculosis encounter.

### Conclusion

The benefits of attending an ART clinic during TB treatment appear multifaceted: ART initiation during TB treatment reduces mortality, and care within an ART clinic reduces loss to follow-up. We therefore recommend that all HIV-infected adults eligible for ART and receiving TB treatment be assessed at ART clinics as soon as possible for initiation of ART. For those with CD4+ counts <50 cells/µL who need to initiate ART within 2 weeks of commencing TB treatment,[5]–[7] immediate (same day) referral for ART assessment and expedited assessment for ART is needed. Early assessment for HIV care by a trained health care provider is also essential. ART initiation during TB treatment reduces mortality and loss to follow-up among HIV-infected adults.
